# On Suicide and Suicidal Insanity
†"On Suicide and Suicidal Insanity, considered in their relations with Statistics, Medicine, and Philosophy." By A. Brierre de Boismont. Paris, 1856. Baillière.


**Published:** 1856-07-01

**Authors:** 


### Part Second.

### Reviews.

### ON SUICIDE AND SUICIDAL INSANITY.†

Is suicide exclusively a medical question? Is it a question that only concerns the civil magistrate? Or is it simply a question of theology or of ethics? At one epoch or another of the world's history, philosophy or theology, the legislator or the physician, has acquired a more or less predominant authority in the solution of this question. In the ages of paganism, philosophy prevailed; since the era of Christianity, religion has exercised the most powerful voice in the decision; to-day, it is medicine that holds the scales. Once, a suicide was a title to honour; then, it was a deadly sin; now, it is a disease. Which is right? One thing is certain—that whether we investigate the phenomena of suicide by the treacherous light of pure philosophy; whether we test them by the precepts of a divine religion; or whether we submit them to medical analysis,—suicide is essentially and above all a psychological question. If, then, we study the subject with reference to its psychological import, bearing in mind the accidental yet numerous and intimate relations with philosophical systems, with religious creeds, with legislative codes, and with medical doctrines, we shall take the surest means of arriving at a knowledge at once broad and impartial, comprehensive and exact. Shall we anticipate the general conclusion to which such a study will lead us? The conclusion will be this: there is truth in all the several tests, if those tests be applied to the solution of particular cases; all are wrong if it be assumed that any one of them is in itself sufficient to solve every case. A suicidist may be sane or insane; and, according to the epoch in which he lived, or the opinions and circumstances by which he was surrounded, he might be a man of exalted virtue or a conscious sinner.

The common sense of mankind, although often clouded by the

† "On Suicide and Suicidal Insanity, considered in their relations with Statistics, Medicine, and Philosophy." By A. Brierre de Boismont. Paris, 1856. Baillière.

changing prejudices of succeeding centuries and of different countries, and often oppressed by the presumptuous dogmas of those who have sought to impose upon the world their own theological interpretations, has always seen through the fallacies of absolute opinions, and has always recognized as real the distinction that strikes the first apprehensions of all between the mental conditions of suicides. Before the era when Christianity shed its gentle light upon mankind,—before erring and simple man had been taught to regard as sacred the image of his Maker, and had learned from the Great Example that the surpassing virtue was to suffer,—the citizen despairing of his country, the man ruined in his fortunes, the defeated warrior, or he who had incurred the displeasure or had exposed himself to the revenge of the powerful, calmly severed the bond that tied them to this life. They thought thus to escape from a world in which their part had been played out, in the same way that by one resolute effort of the will we shake off an unpleasant dream! They were not solicitous for the future; their only care was to flee from present despair. The elysium of the pagan was not shut against him who, weary of the world above, should by his own act seek the companionship of the shades below. The deaths of Lucretia, who would not survive her dishonour, —of Brutus, who could not look upon the ruin of the republic,—of Cato, who would not grace the triumph of Cæsar,—of Cleopatra, who could conquer no more conquerors,—were applauded by men, and represented by the poets as acceptable to the gods. Were these insane? The death of each can be matched in the present day in every noble sentiment; all that will be wanting is the air of grandeur that is derived from antiquity.

The Christian suffers in the belief that this world is a scene of probation,—that he suffers but for an inappreciable moment of eternity,—that endless felicity hereafter is awarded to those who have borne their earthly burden in faith and patience,—and that it is not for him to presume to cut short that stage of trial assigned to him by his Maker. The ancient Roman had none of these motives to respect a life which he regarded as his own, to deal with at his pleasure. It was not, therefore, only in the sublime crises of life—not only when patriotism despaired, when honour was lost, when tyranny was to be baffled—when friends, kindred, or country were to be saved—that the Roman thought it time to die. A ray of heroism that still dazzles even a Christian world hallows the deaths of Curtius, Brutus, and Cato, and tempts us almost to join in the applause of antiquity. These men wanted, indeed, the heroism of endurance, but they were not supported by the faith that animates the Christian. Wanting this faith, they yielded to the noblest impulses of unregenerated humanity. Public virtue and private honour constituted their religion; these sentiments were amongst the most effective safeguards of society; to obey their dictates was to exhibit the loftiest piety and devotion of which the age was capable. We cannot, then, even in the abundant light of the Christian faith, nor in the full reason of manhood, condemn as absolutely sinful, deeds which shone amongst the brightest of olden time, and which still never fail to evoke the first generous emotions of classical boyhood.

And although the progress and spread of civilization and of true religion have, since the epoch when the Roman Empire represented the whole civilized world, been great, that progress and that spread have still been partial and unequal. Therefore it is that we may, even at the present day, by simply looking around us and surveying the existing world, discover living types of all those conditions of barbarism, systems of philosophy, and theological creeds, which have successively, at some period or another, stamped the character of an age. If we avail ourselves of this fact, we shall find that we possess all the advantages that actual observation can bring to the study and elucidation of this difficult question. If, for example, we wish to inquire into the mental condition of the patriot or the warrior who thinks it glorious, or at least not infamous, to die by his own hand when he despairs of his country or of military success, we need not turn back the pages of history to search for instances; we may examine recent cases which have all the completeness of detail and all the instructiveness that clinical demonstration in the wards of an hospital possesses, as compared with the reading of the medical observations recorded by ancient physicians.

In the accounts we receive of the remarkable and mysterious civil war that has for some time past been devastating China, we constantly hear of defeated generals and soldiers who voluntarily sever their connexion with a world in which they could not prevail. India, again, the land of mystic philosophy, still teems with examples of suicides that bear all the characters of acts of devotion, and which we cannot attribute to the influence of insanity, unless we are prepared to maintain that the entire philosophy and religion of the Brahmins and Hindoos are but phenomena of insanity also. The "Bhagavad-Gita" inculcates doctrines and precepts which lead to the annihilation of will; which, in their ecstatic sublimity, seem to lift their votaries out of the material world, and which reduce self-murder to an act of indifference or of fate:—

> "Wert thou loaded with sins, thou mightest pass the abyss in the bark of Wisdom. Know, Ardjouna, that as natural fire reduces wood to ashes, so the fire of true wisdom *consumes all action*. . . . . The presumptuous man believes himself the author of his actions; but all his actions spring from the necessary force and concentration of things."

Under such a creed, suicide is nothing more than the revolution of the hands of a watch which has been duly wound up. It implies neither criminality, nor sin, nor disease. What it does imply, is utter moral darkness, and the deepest intellectual degradation. If we would estimate the mental conditions and the motives which led the Stoics and Epicureans of ancient times to self-murder, we are not reduced to the contemplation of historic examples. In the bosom of modern civilisation are nursed men who, by their scepticism concerning religion, future life, or posthumous rewards and punishments, may be studied as the representatives of those who adopted for their maxim, "Mori licet cui vivere non placet." Do we believe that Seneca was mad, or Diogenes or Zeno, or Lucretius or Diodorus? No; in judging the deaths of these philosophers and historians, we take into account the nature and tendency of their philosophical and theological doctrines. Lucretius formulised his creed in the following words:

"Nil igitur mors est, ad nos neque pertinet hilum;
Quandò quidem natura animi mortalis habetur!"

Plato, in the depth or the sublimity of error, thus expounded the liberty and the restrictions of suicide: "He is not to be blamed who kills himself, unless he does so without the authority of the magistrates, or without being driven to it by a painful and intolerable position, or by the dread of a future filled with misfortunes." Pliny, in the arrogance of human pride, was not content even with this limitation. He professed to see in the power which man possessed, through the gift of reason, to leave this world at his pleasure, a mark not only of his superiority over all other created beings, but even over the gods themselves: "Imperfectæ verò in homine naturæ præcipua solatia, ne deum quidem posse omnia. Namque nec sibi potest mortem consciscere, si velit; quod homini dedit optimum in tantis vitæ pœnis." And Cicero, whose timid nature, more, perhaps, than his reason or his sense of right, often restrained him, under the reverses and mortifications of his latter days, from carrying into effect a wish he no doubt experienced, has thus, whilst admitting a divine injunction against suicide, placed it in the power of every one to interpret the divine will at his pleasure: "The God who holds over us a sovereign power, will not allow us to quit this life without his permission; but when he has caused a just desire to do so to spring up within us, then the truly wise man should pass with pleasure from the gloom of this world to the celestial light." Are there not still men amongst us who hold opinions not essentially different from these? and is not suicide a logical consequence of such opinions? Take, for example, the death of Philip Strozzi. In the suicide of this man we witness the conflict of a mind in suspense between Christianity and the dogmas and examples of the heathen philosophers. Taken prisoner by the Grand Duke Cosmo I., and accused of participating in the assassination of Alexander I., he destroyed himself in order to avoid compromising his friends under the influence of the torture. This fragment of his last will would have been held up to the admiration of the ancient world; it excites a feeling of pity rather than of condemnation in the Christian heart; neither then nor now does it bear evidence of insanity:—

"To the liberating God. To remain no longer in the power of my barbarous enemies, who have unjustly and cruelly imprisoned me, and who might compel me, by the violence of tortures, to reveal things hurtful to my honour, to my friends, as has happened recently to the unfortunate Julian Gondi; I, Philip Strozzi, have taken the only resolution left to me,—however fatal to my soul it seems to me,—the resolution to put an end to my life by my own hands. I

recommend my soul to God, a merciful Sovereign, and humbly pray him, as the least grace, to accord it, for its last dwelling, the region where dwell the souls of Cato of Utica, and of those virtuous men who have made a like end."

The history of philosophy may, perhaps, be considered by the Christian divine or the Christian physician as the history of human error; but neither will so far surrender his judgment to his religious or pathological preconceptions as to regard the history of philosophy as the history of sin or of mental alienation. If this be granted,—if we attribute the voluntary deaths of such men as Zeno, Seneca, and Diodorus to the influence of a false philosophy,—how can we refuse to attribute the deaths of such men as the Girondins, Pétion, Barbarossa, Roland, and Condorcet, to the same cause? Did all false philosophy, all error, disappear from the minds of men with the birth of Christianity? Or has the cerebral organization of man changed since the days of pagan philosophy? Is that same subjection to those doctrines which inculcate it as a right, if not a duty, to commit suicide, to be cited as an illustration of a peculiar philosophy in an ancient Roman, and as a proof of disease in the modern sceptic? No; the common sense of mankind rejects these absolute and special conclusions. Howsoever pure we may esteem our own religious faith, however true our scientific knowledge, we cannot but recognise the fact that false religion and error have been kept alive in all ages, and still exist in the heart of the most refined disciples of modern civilization. Is not the couplet of Voltaire something more than a mere verbal translation from the olden philosophers? is it not the confession of faith of many a modern sceptic?—

> "Quand on a tout perdu, et qu'on n'a plus d'espoir,
> La vie est un opprobre, et la mort un devoir."

To pronounce, then, as did the mediæval priest, that all suicide is sin, or to contend, as some modern physicians, jurists, and others do, that all suicide is disease, is to worship the *idola Specûs*—to hold up between our eyes and the object to be observed a refracting and discolouring medium, that presents a false image to our perception, and thus destroys the foundation of accurate judgment.

But it is not only in causing suicide that false opinion acts. False opinion has, in all ages, led to the commission of other acts scarcely less to be condemned by sound reason. The assassination of Julius Cæsar sprang from the same exalted patriotism as the suicide of Brutus; but it is not pretended that the assassination was the act of a madman. Like moral perversion, like social and political doctrines, will lead one man to murder his fellow-man, another to sacrifice himself. To single out suicide from the list of offences, to assign this alone to insanity, and to attribute the rest to false opinion, is an arbitrary distortion of the truth, a wanton inconsistency that prejudges the whole question, and defies all argument.

We have gone thus far in the exposure of what to many must seem an absurdity too palpable to need refutation, because it is impossible to observe the passing history of daily life without being struck by the growing disposition evinced by coroners' juries to bring in verdicts of "Temporary insanity" in every case of suicide. There can be no doubt that, in a country more or less generally illumined by the light of the Gospel, and guided by modern science, suicide is more frequently the result of cerebral disease than it was in olden times. The proportions of suicides through false opinion and disease are doubtless changed. In ancient times, the number of suicides from disease was small compared with that of those suicides which flowed from the prevalent philosophical doctrines. In our times, the presumption in favour of disease, as against false opinion, rises to a much higher degree. But still the same elements exist, and will continue to exist.

We cannot but rejoice that the author who has produced a work based on the analysis and synthesis of the largest number of facts, and marked by the most comprehensive survey of the moral, intellectual, and physical relations of suicide, has deliberately recognised this broad distinction between the suicide of the madman and the self-murder of the responsible being. M. Brierre de Boismont has, in his very title, announced his conviction upon this fundamental point. By the words "On Suicide and on Suicidal Insanity," he clearly traces the line of demarcation, placing on the one side the sane man, who is responsible, and on the other side, in distinct opposition, the madman, who is not. In an historical introduction, he takes a critical survey of the influence of the prevailing social, political, and moral conditions of mankind in producing or discouraging suicide in ancient, middle, and modern times. In this survey we cannot follow him minutely. The practical lesson which it teaches we have indeed anticipated in the preceding reflections. His summary is all that we need here extract. Antiquity, by its philosophical and religious doctrines, all essentially pantheistic, was favourable to the development of suicide. The middle ages, on the contrary, by the establishment of the Christian religion, by the predominance of the religious feeling and of the spiritual philosophy, succeeded in checking the progress of this evil. Lastly, modern times, by propagating doubt, exalting pride, making out of self-love, scepticism, and indifference, a sort of code for the use of the many, have given a new impulse to suicide.

But while recognising implicitly as a fundamental truth the absence of all necessary connexion, as cause and effect, between insanity and suicide, M. de Boismont gives a prominent place in his etiology to those transmitted somatic peculiarities which are easily traced throughout families, races, and nations. In speaking of the French, he says:—

"Of all modern peoples, there is none in whom the general sensibility, the link of union between the world of facts and the world of ideas, is more developed than in the French. Of prodigious mobility, passing from one extreme to the other, giving birth to prodigies, and presenting the spectacle of the most abject miseries; braving the greatest dangers, and annihilating itself to save life and fortune; possessing in the highest degree the courage of the warrior, and almost completely destitute of civil courage; dragging in the mire what it worshipped yesterday; seeking emotions even in the refinements of death; witty, gay, generous—then wearied of wit, pleasures, and charity; coveting everything with avidity, and changing in an instant ideas, thought, and will; ever led away by sentiment, the Frenchman presents in himself all that characterises the qualities and the defects of general sensibility. Hence it is easy to conceive why suicide is so frequent. To what is this predominance of the sensibility in our country owing? Is it to the action of races, to the drop of original blood, the influence of which has been disputed? Does not the vital afflatus transmitted by parents, that evades all our instruments of research, contain in its subtle essence the features, the virtues, the vices of families?"

Thus, in our very first steps in the investigation of the causes of suicide, we find ourselves linked with the past by the chain of succeeding ages through heredity; an indubitable proof that if man is, on the one side, a new individuality by the creative force that is in him, he is, on the other, the continuation of his race, of his family, of which he represents a certain evolution.

The great fact of heredity in moral and intellectual character bears a direct and important relation to the etiology of suicide. But, being one of those great facts which all mankind admit, it is the more imperative, in a scientific investigation, not to suffer ourselves to be unduly swayed by it, not to discover in it the ready and only solution of the problem. To show the error and danger of hasty generalization in this matter, the case may be stated as follows:—The hereditary transmission of mental peculiarities is an undoubted fact; it is also an undoubted fact that mental peculiarities are closely connected with physical peculiarities of organization hereditarily transmitted; further, it is frequently observed that the disposition to suicide is transmitted through several generations, or at least it is an undoubted fact that in numerous instances the ancestors or consanguineous relations of suicidists have also committed or attempted suicide; again, it is also a fact established by the records of our lunatic asylums, that the ancestors or other consanguineous relations of the insane have in many instances committed or attempted suicide. Now, the simple consecutive statement of these undeniable facts tends at once to suggest to the mind the conclusion that, since the disposition to suicide is so frequently found in hereditary association with insanity, suicide itself is therefore a manifestation of insanity. But a large survey of facts and a close analysis will prove that this conclusion is only partially true, that it explains the etiology of a certain portion only of cases of suicide. To admit it absolutely, is but to admit in another form the doctrine of the "Bhagavad-Gita"—the doctrine of necessity. The logical fallacy of the argument will be evident to all who will submit it to analysis.

But we will admit at once what there is of truth in this argument, and assign a high place to the influence of abnormal cerebral organization or central disease in the production of suicide. We possess ourselves of this fact as explaining a very large proportion of the cases of suicide that occur at the present day. We pass on to other examples in which the influence of disease of the brain may be inferred if not proved, or in which at least the effects of exhaustion, and of the absence of that nutrition by which the healthy action of the brain can alone be sustained, is obvious. Under the influence of long-continued physical want and moral afflictions, it is certain that the brain, like every other organ of the body, is liable to suffer such a waste or atrophy of its substance that it becomes unable to perform its functions efficiently. The mental phenomena, the brain-symptoms, of advanced stages of scurvy are as remarkable as the visible bodily symptoms. The starved brain has lost its vigour, the mind is without energy, and if in such a state the unhappy patient refrains from suicide, it is because a torpid indifference to life and to everything besides has displaced all will and power to act. In cases where the dyscrasia is less advanced, but where the moral torture is greater, the impulse to self-destruction has, however, been remarkably manifested. The horrors endured by our brave army in the Crimea will occupy a large space in the narrative of the historian, and his long chapters of misery and despair will ever arouse the pity and indignation of posterity. Yet, let him not spare the last scene in the most frightful tragedy of modern times; let him not suppress one count in the damning bill of indictment against those who have to answer for the destruction of a noble army which they undertook to lead to victory. We will here supply a fact which we believe has not been made known to the public, but which, more than any other that has been recorded, marks the abject depth of the misery into which that army was sunk by titled recklessness and official idiocy. Men in the hospitals, prostrated by scurvy, all physical energy lost for want of nourishment, and with despair in their minds from the maddening conviction that the incapacity of their commanders held out no hope of relief, earnestly protested that they were convalescent, begged to be discharged as fit for duty, and entreated to be sent to the trenches, in the hope—the only hope that was left to them—that there they might meet with the death they prayed for, as preferable to the horrors of life. It was not the delirious wish to join their comrades in service against the foe that kindled up for a moment those exhausted and expiring men; their fixed and only thought was that, once laid down in the trenches, exposure, cold, wet, accident, a shell, or what not, would soon release them from this world. How near akin is this to suicide!

It might be that a sense of religion sustained these wretched men so far as to save them from self-destruction by the direct agency of their own hands. It might be that this mental and physical prostration was so great, that they had not the power to accomplish what they longed for. But how near were the feelings of these men to suicide, may be illustrated by the fate of men of a different race under analogous circumstances. It is related that, in the construction of the railway across the Isthmus of Panama, a large body of Chinese were employed as navigators; that, under neglect, rapacity, and barbarous treatment, they became indifferent to life; and that, when they saw many of their comrades carried off by fever, the desire of death spread like another epidemic amongst them, and they destroyed themselves in great numbers by ripping up their bellies, or by lying down on the sea-beach at low water, so that the flood-tide might drown them. Between this latter mode of inviting death and that coveted by our soldiers in the trenches, where is the difference? What, again, is the difference between the case of the man who thus puts himself in a position where death is certain, and that of him who throws himself from a precipice, knowing he will be dashed to pieces at the bottom? It is simply a difference of time and mode. It is active suicide in both cases.

From cases of this kind, in which physical and moral causes concur to impair the body and disorder the mind, let us consider for a moment other cases in which the influence of moral suffering and of intellectual self-exulceration is clearly greater than that of physical distress. The case of the political prisoner is perhaps one of the most instructive in this respect. Although the want of variety of diet, of pure air, and of healthy exercise, cannot be eliminated from the inquiry, it is certain that isolation and the attendant moral and intellectual penalties of long captivity exert a far more potent influence in the production of insanity and suicide. We dwell for a moment upon this subject, because it is in a right psychological and medical aspect of it that we may learn to understand better some of the bearings of solitary confinement, and draw useful lessons in the treatment of prisoners and of the insane. We all remember that touching anecdote in the "Spectator," of a gentleman who had been under close confinement in the Bastille for seven years. During this time, this gentleman amused himself in scattering a few small pins about his chamber, gathering them up again, and placing them in different figures on the arm of a great chair. He often told his friends afterwards, that unless he had found out this piece of exercise, he verily believed he should have lost his senses. Something similar is told of Francesco Madiai, who preserved his reason when confined in prison in Tuscany for his religious opinions. But a more remarkable and instructive example, because it exhibits the mental struggle of one of the most gifted and commanding intellects of the age, is that of the great Hungarian patriot:—

"For months I was there, in a damp, lonely chamber, seeing neither the sky nor the earth, with none of those inexhaustible consolations which bountiful Nature affords to misfortune and suffering. And there I was, without a book to read, without a pen to write; there I was with God, with my tranquil conscience, and with meditation alone. But it is fearful to be thus alone, with nothing to arrest the musing eye. Imagination raises its dreadful wings, and carries the mind in a magnetic flight to regions of which no philosophy has ever dreamt. I gathered up all the strength of my mind, and bade it stop that dangerous soaring. It was done; *but I got afraid of myself*. So, I told my gaolers to give me something to read. 'Yes,' answered they, 'but nothing political.' 'Well, give me Shakspeare, with an English grammar and a dictionary.'"*

What a vivid picture of the danger of solitude upon the human mind! We feel that a mighty intellect rocked on the verge of

* Speech of Kossuth, May, 1853.

insanity. We feel that he was only saved from suicide by communion with the spirit of our immortal bard! Under similar circumstances, how many unhappy men have actually succumbed! But here, again, we must appeal to analysis. Not all even of those who perish by their own hands in prison are insane. The old Roman, in the position of Kossuth, would probably not have waited for the advent of disease. So, in like manner, may some in the present day release themselves from the thraldom of this world on the first impulse of despair. Although altered customs and modes of thought, and above all, the spirit of submission inspired by Christian sentiments either actually possessed or reflected upon them, preserve the greater number of prisoners in our times from suicide during the predominance of reason, it cannot be doubted that some make the attempt, and that some succeed. An extract from M. Ferrus's philosophical work, "Des Prisonniers, de l'Emprisonnement et des Prisons," will confirm this conclusion. This eminent physician says: "There are no suicides, or at least they are excessively scarce, amongst female prisoners, although it has been seen that *more cases of insanity* are developed amongst them."

We may here introduce some facts showing the influence of imprisonment in causing suicide in France. From the researches of M. Pietra-Santa, it appears that there occurred between 1850 and 1854, out of 25,268 prisoners at Mazas, 24 suicides and 43 attempts; in the Vieille Force, between 1840 and 1849, out of 37,397 prisoners, 3 suicides and 4 attempts only.

But it is time to make our readers acquainted with some of the more striking and best authenticated of the conclusions of M. de Boismont respecting suicide in France. Various statistical tables relating to France and Geneva all concur in showing that suicides in females bear about the proportion of one-third only to those in men. It has also been found that suicide is extremely rare in children under the age of fifteen; but not so rare, but that M. de Boismont is struck with the enormous disparity between the frequency of suicide and that of insanity in children. Excepting the cases of idiocy and epilepsy, he has observed but two cases of insanity in children out of two thousand insane patients. There is, he says, a decided line of demarcation in youth between suicide and insanity; and this appears to depend, in a certain degree, upon the different pathognomonic conditions of these two states. The greatest number of suicides take place in Paris between the ages of twenty and thirty; in the departments, between forty and fifty. It results that celibacy, both in men and women, exhibits a greater proportion of suicide than the married state.

The influence of education is a question surrounded with difficulties. The vulgar test, the one too often relied upon in our criminal statistics at home, is the ability to read and write. But it is impossible to deny that there are many persons who can neither read nor write, who yet possess more real education, better mental training, and more knowledge, than thousands who can do both. Tried by this test, it was found that out of 4595 male and female suicidists, 1362 could read and write well; that 1656 could write, but without orthography; that 3

could read, but not write; that 65 could do neither; and that for 1509 no information was obtained. Our author gives a detailed table of the station in life and occupation of persons who committed suicide; but as we are not in possession of accurate census returns exhibiting the absolute or relative numbers of persons engaged in different trades in France, this table can convey no definite notion as to the influence of the various employments. He adverts, however, to the excessive proportion of artisans. One illustration, which we owe to the historical researches of M. Sainte-Fare Bontemps, is so curious that we introduce it here. This author has found that out of 2542 chiefs or sovereigns belonging to sixty-four countries, 20, or 1 in 127, committed suicide; and 11, or 1 in 221, became insane.

M. de Boismont has analysed the reports of 4595 cases of suicide, in such a manner as to exhibit, in a tabular form, the presumed causes of this catastrophe. We must, however, premise that we attach much less importance to this table than does the author. The assigned cause is, in most cases, simply the obvious or presumed immediately exciting cause. To discover the real cause, we must go back further in the history of the victim's life; we must know more of his physical organization, more of his psychical *indoles*.

Presumed causes of suicide in 4595 cases:—

| | | | |
|---|---|---|---|
| 1. | Drunkenness | 530 | 1309 |
| | Poverty, misery | 282 | |
| | Pecuniary embarrassment, reverses of fortune, cupidity | 277 | |
| | Misconduct | 121 | |
| | Idleness | 56 | |
| | Want of work | 43 | |
| 2. | Insanity | 652 | 1089 |
| | Ennui, weariness of life | 237 | |
| | Feeble, exalted, sad, or hypochondriacal character | 145 | |
| | Acute delirium | 55 | |
| 3. | Domestic griefs | 361 | 672 |
| | Griefs, disappointments | 311 | |
| 4. | Diseases | 405 | 405 |
| 5. | Love | 306 | 360 |
| | Jealousy | 54 | |
| 6. | Remorse; fear of dishonour, of legal prosecutions | 134 | 134 |
| 7. | Gaming | 44 | 44 |
| 8. | Pride, vanity | 26 | 26 |
| 9. | Various motives | 38 | 38 |
| 10. | Unknown motives | 518 | 518 |
| | | Total | 4595 |

Analysis of this table will at once show that the purely moral causes, as distinguished from those moral conditions which are associated with physical disease, are greatly exaggerated. It would convey but a very imperfect idea of the influence of insanity, if we were to assume that the proportion of cases of suicide due to this cause is accurately represented by the figures in this table. If we add together all the cases of class 2, we have 1089 cases out of 4077 (excluding the 518 cases, cause unknown) or about one-fourth only assigned to mental alienation—a proportion, we hesitate not to say, very much below that which observation justifies. We are not amongst those who are led away or dominated by what are called statistical tables. A statistical table, to be worthy of the name, requires that the constituent facts shall have been rigidly sifted, and accurately recorded and classed. The proper use of a statistical table is to *lead to* the discovery of aggregate facts or general laws. But when, as in the case before us, a table is constructed according to a predetermined theory, the general laws are anticipated, not deduced; and whether or no the table represent facts, that is truths, at all, is a matter of accident. Who, for example, can estimate the number of cases in this table classed under the heads of drunkenness, misconduct, diseases, that might not with more justness be transferred to insanity? Where a rigid and exact observation has not governed the synthesis, the construction of a statistical table, then a rigorous analysis must be applied to the table, and the probability is, that under this process it will crumble to pieces again, and be resolved into its individual elements. And this, in fine, is the only course open to the scientific critic in the present case. If we would determine the truth of M. de Boismont's theory, that there is a distinction between cases of suicide from insanity, and cases without mental alienation, we must distrust the inference from his table, reject as unproved and incapable of proof the proposition which states the proportion of irresponsible suicides to the responsible to be as one to four, and seek for the truth by the observation and study of particular cases. The proportion of one insane in seven suicides, given in another place (p. 139), is of course a still further divergence from the truth.

We shall presently cite some examples in proof of the proposition that sane men may commit suicide. We dwell for a moment upon some of the conditions of mental alienation which lead to this act. The influence of imitation is a very remarkable phenomenon, and is well known. Without stating absolutely that in every case of imitative suicide the person was insane, we believe that the exceptions are rare, and that even amongst these exceptions we may trace evidence of defective cerebral development, if not of actual disease. It is obvious from the fact that many of those feeble or diseased organisations yield, as if to an irresistible power, to the fascination of example, are as ready to sacrifice themselves to one example as to another: they hang themselves because others have done so; they throw themselves from the Monument because a recent example suggests to them that mode of self-destruction. Wanting such examples, they would probably discover some means by themselves, or their insanity would become manifest in other ways. It may be true that when a soldier having killed himself in a sentry-box, other soldiers selected the same sentry-box for suicide, imitation was arrested by burning the sentry-box; or that in the Invalides, a pensioner having hanged himself in a doorway, twelve men hanged themselves within fifteen days at the same place, and that this rage for hanging was stopped by bricking up the doorway. It is not a logical inference that these imitators were sane men suddenly wrought upon by the force of example. It would be necessary to search their antecedent history; this would probably afford a clearer explanation of their conduct.

We have already seen that suicide is comparatively rare in women; but another fact in female suicide is also established, namely, that the greater proportion of cases take place within the ages of fifteen and fifty-five. Now, it will be observed that this period is precisely that of the preponderance of the reproductive system. We believe that the presumption is very great indeed, that any case of suicide happening within this epoch, is directly associated with some disorder of the blood and of the nervous system, arising from permanent or temporary morbid conditions of the sexual organs. We are especially confirmed in this opinion by the experience of Dr. Barnes, a physician who is not only well versed in the study of mental alienation, but who, by his extensive observation of the diseases of females, enjoys peculiar opportunities for studying their morbid psychological symptoms.

The influence of pregnancy and of parturition is familiar to all, although not, perhaps, sufficiently understood in all its bearings. In the tables of M. de Boismont, we find no less than twenty-seven of the women who committed suicide were pregnant. Menstruation, especially some forms of abnormal performance of this function, is often attended by states of mind that cannot be distinguished from temporary mental alienation—states in which vertigo, despondency, hallucinations, and suicidal or homicidal ideas harass the patient, and make her afraid of herself. But it is especially at the critical age, at the decline of the menstrual function, that these symptoms are exhibited in all their intensity. They are then frequently attended by attacks of delusional and maniacal hysteria, hysterical paralysis, and even epilepsy. In many cases, such patients retain sufficient moral control to dominate their morbid thoughts and impulses, sometimes they conceal them altogether from those around them, but sometimes it is certain that they succumb to their malady.

The forms of alienation which have the greatest influence in the production of suicide, are: monomania, with depression; exaltation and mania may lead to suicide, through the illusions which accompany these states; hallucinations; general paralysis, occasionally; and sometimes an apparently sudden access of insanity.

But in tracing the history of suicide, and describing the various mental phenomena associated with it, it is impossible to pass over a remarkable form of mental alienation which may be discerned by its peculiar features through all the ages of civilisation. Seneca has thus described this disease:—

"The evil which oppresses us is not in the spot which we inhabit, it is in ourselves; we are powerless to bear anything, incapable of enduring pain, impotent to enjoy pleasure, impatient of everything. How many call upon death, when, after trying every change, they find themselves plunged again in the same sensations, without being able to experience anything new! Life, the world, is a burden to them; and in the very bosom of luxury they exclaim, What! always the same thing!"

This disease had a name derived from the Greek—*athumia.*

In the time of Seneca, suicide seems to have been a true contagious disease. Christianity modified, but did not remove this affection. The cloisters often became the refuge of its victims. No psychological physician of the present day could paint it more vividly than has been done by St. Chrysostom and St. Jerome. The latter describes a different form of the disease, clearly arising from bad hygienic and moral modes of life:—

"There are monks," he says, "who, through the dampness of their cells, immoderate fastings, disgust of solitude, excess in reading . . . . fall into melancholy, and who want the remedies of Hippocrates rather than advice from me. . . . I have seen persons of both sexes whose brain had been affected by too much abstinence, especially amongst those who dwell in cold and damp cells. They no longer knew what they did, nor how to conduct themselves, what to say, or what to keep silent."

The condition here described by the ecclesiastical historians of the middle ages assumes more distinctly the characters of hypochondria and lypemania. They gave it the name of *accidia.* Cesarius relates some examples in his "Dialogus Miraculorum," composed in the thirteenth century. The following is one:—A nun of advanced age, of exemplary piety, becomes all at once troubled and tormented by the evil of melancholy—the spirit of blasphemy, of doubt, and scepticism. She falls into despair—refuses the sacraments; then, believing herself condemned to eternal fire, and fearing that, according to the threat of her confessor, the prior, her body will be buried without honour in the fields, she leaps into the Moselle.

The "Speculum Morale" of Vincent de Beauvais contains the following graphic description:—

"Accidia est quedam tristitia, aggravansque ità deprimit animam hominis, ut nihil ei agere libeat, et imo accidia importat quoddam tedium benè operandi . . . . Filie accidie multe sunt, quod multis modis per accidiam peccat homo. Ejus autem filie sunt hec: dilatio, signities, sive pigritia, pusillanimitas, inconstantia sive imperseverantia et inquietudo corporis, evagatio mentis, ignorantia, ociositas, verbositas, sive multiloquium, murmur, taciturnitas mala, indiscretio, gravedo, tedium vitæ, impeditio bonorum, impenitentia, desperatio . . . ."

These writers speak with a kind of horror of these cases, as if it were a scandal to the religious houses that they should have occurred; they scarcely considered the sufferers as diseased, but rather as being deeply guilty. The revival and spread of the sensualist doctrines in the eighteenth century gave a new turn and new features to that stock of *ennui*, or melancholy, which has probably never ceased to afflict the human race. The religious aspect gave way to one of an opposite kind. Rousseau, in his "Saint-Preux," Goethe in "Werther," depicted the new features of this mental disorder. And, in the present century, the "René" of Chateaubriand, the "Raphael" of Lamartine, reproduced scarcely different types of the same disease. Each of these characters of romance is led by the author to the contemplation of suicide.

M. de Boismont contends, in opposition to Esquirol, that insanity has no part in these suicides from *ennui*—from melancholy.

"The ideas," he says, "education, and doctrines of the time perfectly account for this condition of the mind. It is by no means necessary to be mad in order to be gnawed at the heart, at the present epoch, by *ennui* and weariness of life. When no one is sure of the morrow; when reputation, property, fortune, have nothing stable; when Conservatives and Socialists begin all their writings with this phrase—*We travel towards the unknown;* when, looking around us, we discover nothing but ruins—not an institution standing; when intellect seeks for shelter beneath the sword;—do you believe that the tranquillity of soul of which Seneca speaks is at the command of the greater number? This foreboding of approaching evil, is it not general? In seeing the people rush like torrents in search of pleasure, do we not understand that they seek to fly from themselves, and turn aside their eyes from the ill that is at their doors? Is it not the faithful image of the Jews at the siege of Samaria, crying out, 'Let us drink and eat, for we shall die to-morrow?'"

We have only to remark upon this painful picture of the state of feeling in Paris during the anarchy of 1850, that however true it may be as explaining the recklessness of life and disposition to suicide there at that time, it by no means accounts for the perfectly analogous phenomena which have at all times, and in all countries which history or observation informs us of, existed, although, perhaps, sometimes in unequal extent. The perpetuity and universality of these sentiments and propensities bespeak a more inherent, more profound, and more intimate relationship to the physical organisation of man than M. de Boismont represents.

Out of the 4595 reports which have served for the foundation of M. de Boismont's researches, the number of notes, letters, scraps of verses, or other documents, left by those who destroyed themselves through *ennui*, disgust, despair, scepticism, indifference, materialist convictions, amounts to 237. They are divided into two series: the first comprises those in whom spleen, *tedium vitæ*, succeeded to some grief or suffering—secondary or acquired *ennui*; the second, those suicides in whom reverie, melancholy, always existed—original, primitive *ennui*. It is obvious that the second class, at least, of original *ennui* can hardly be accounted for on any other supposition than that of being connected with primitive somatic peculiarities. It would probably be more correct to add these cases to the list of the insane.

To the same list must be added a very large proportion of those who, in M. de Boismont's classification, are described as having committed suicide in consequence of disease. The following passage is entirely in accordance with our own experience, and reflection upon its import might have satisfied the author of the error of his classification:—

"There is an organ whose sufferings appear to have a marked influence on suicide,—that is, the stomach. The predominance of sad ideas in those who digest badly has long been observed. Chronic gastritis, gastralgic affections, cancer, predispose to sadness, melancholy, suicide, insanity. Without denying the part of the brain in hypochondria, it must be admitted that this disease often has its point of departure in the stomach, intestines, and ganglionic system. As soon as hypochondriacs cease to suffer, sad ideas vanish as if by enchantment. Another remark which we have many times had occasion to make, is that the gastralgias which had brought about serious perturbations in the digestive functions alternate with mental diseases, and that on the appearance of insanity all the disorders of the digestive functions cease."

We might pursue this analysis much further, but we have shown enough to prove that an immensely larger number of suicides than that stated by M. de Boismont must be transferred to mental alienation as the true cause.

A point upon which it is of deep interest to entertain clear ideas, is whether we ought to recognise a distinct form of insanity, of which the disposition to suicide is the pathognomonic character. If it be true in nature that suicidal insanity, as a species or type apart, exists, it is exceedingly important to establish on clear pathological facts and demonstration. A careful analysis of particular cases—not a statistical conglomeration, which, for the most part, has no other effect than hiding from our sight that clear knowledge which special clinical observation supplies—will enable us to solve this question. This analysis will speedily prove one fact,—namely, that a first case of suicide occurred in a monomaniac afflicted with illusions, that a second occurred in a maniac during a paroxysm of frenzy, that a third occurred in a patient afflicted with general paralysis, that a fourth occurred in a confirmed hypochondriac, and so on, until we arrive at the positive discovery that cases of suicide are observed in any and every form of insanity. We trace the particular histories further back, and we discover that in many of them the disposition to suicide was a development subsequent upon other manifestations or symptoms of insanity: rarely, indeed, if ever, will it be established that suicide sprang up in the mind as the original, essential symptom of the disease. Having gone thus far in the analysis, it will be found that we have eliminated by far the greater number of cases of suicide, or suicidal propensities, from the inquiry. In the immense majority it will be seen that suicide and suicidal thoughts are but an epiphenomenon, a symptom of some well-known form of insanity. There remain a small class of cases which, as they are more difficult of analysis, occasion greater difficulty in the endeavour to assign them their true place. These are the cases of sudden suicide in which little or no antecedent account of the victim's true somatic and psychical condition can be obtained; these are the cases analogous, at least in their appearance, to impulsive homicidal insanity, as it is called. Now, it has been clearly proved, for many of these cases of so-called impulsive homicidal insanity, that some marked bodily or mental disorder existed for a period more or less prolonged, before the manifestation of the homicidal act or attempt; and in others of these cases, subsequent observation of the patients has furnished demonstrative evidence of the existence of some well-recognised form of insanity. In other cases, again, it may be fairly doubted whether there was any insanity at all,—that is, whether they were not purely cases of murder. The application of this analogy to the assumption of impulsive suicidal insanity is perfectly legitimate. We are entitled to conclude that more minute history of the antecedents, and more frequent opportunities of observation after the suicidal attempts, would reveal distinct evidence of some one of the common forms of insanity. In some few cases, moreover, it would be perfectly arbitrary to assume that there was any insanity at all. The cases, then, which seem to favour the theory of a specific suicidal insanity, dwindle down to an evanescent point, and scarcely leave enough of fact whereupon to base an argument.

A survey of the modes of death resorted to by suicidists will supply numerous interesting illustrations of the different habits of thought, customs, laws of different countries and classes, and, in some cases, throw considerable light upon the mental condition and form of mental alienation of the victim. This is also the point which most deserves to arrest the attention of the medical jurist.

The different modes of self-destruction observed in Paris are classed as follows:—

1. Asphyxia by charcoal.
2. Drowning.
3. Strangulation.
4. Fire-arms.
5. Precipitation.
6. Cutting instruments.
7. Poisoning.
8. Crushing.
9. Abstinence.

Other returns for the whole of France place submersion and strangulation at the head of the list.

The mode by asphyxia is especially resorted to by women. This circumstance is accounted for by the universal use of charcoal for domestic purposes in Paris, and the consequent familiarity in the management of it. If we compare the favourite modes adopted in England with what is observed in France, we shall find some remarkable differences. We must in this place premise, that generally excellent as is our system of registering the causes of death, and its admirable administration by that accomplished statist, Dr. Farr, it has failed to furnish us with the same amount of detailed and complete information upon the subject of suicides which is collected in France. Several causes concur to impede the collection of trustworthy information in England. The inquisitions of coroners' juries are often, nay, in most cases, conducted with much inferior minuteness and care than are exercised in France, where an *expert* is charged, in every case, to make a full report. With us, too, there is a disposition amongst coroners' juries to seize upon the most trivial circumstances in order to make a colourable justification for a verdict of "Temporary insanity." The parsimony, and often culpable indifference, of county magistrates, too, by refusing the necessary funds for conducting efficient and skilled investigations, frustrates all hope of ascertaining the cause of death in many cases. The vain, if not blasphemous verdict, "Died of the visitation of God," is still, in many parts of the country, the refuge of ignorance, indolence, or indifference. Under this, or some other vague term, is doubtless concealed many a death by suicide or by foul murder.

We must therefore abandon the desire to present any extended comparative view of suicide in the two countries. We commend these considerations to our author, who, in his love for statistics, has overlooked here, as in other instances, grave defects in the collection of the individual facts, such as must render many general conclusions utterly fallacious. The determination of the relative frequency of the various modes of death is, however, one thing which the facts in our possession enable us to effect. Out of 232 suicides in this country, there were

91 cases of hanging, cutting instruments were used in 47, poisoning 45, drowning 29, precipitation 10, and fire-arms in 10 cases. Asphyxiation, then, so common in France, is unknown amongst us—a circumstance obviously accounted for by the little use of charcoal. It is deserving of especial remark, that poisoning rises to a much higher rank in England. M. de Boismont notes 157 cases only of poisoning out of 4595, whereas in England the cases of poisoning are quite one-fifth of the whole. How is this explained? Also very easily. In France the sale of poisons is restricted by the most vigilant legal provisions. Here nothing is so simple as the obtaining poison enough for any purpose of suicide or murder. It may be said that this makes little difference in the result; that in France, where poison is difficult to obtain, charcoal is always at hand; that the commission is in no degree checked or encouraged by the restriction or freedom of the sale of poisons; that, in short, those bent upon suicide, failing in one means, have only to select another. Even as regards suicide this is not altogether true; the slightest delay or disappointment in compassing the means first sought may save the intended victim from himself. But in relation to the facility of obtaining the means of committing murder, there cannot be a doubt as to the necessity of imposing the most rigorous restrictions upon the sale of poisons.

The influence of season upon the production of suicide in France seems to be remarkable. Thus, in ten years the highest figures are observed in the months of May, June, and July; the lowest, in November, December, February, and January. Grouping the months in series of four, the following result is obtained: out of a total of 4595, there happened in the first four months of the year 1491, in the second term 1837, and in the last four months 1267.

M. de Boismont's figures show an annually-increasing number of suicides in France and in Paris; he fails in demonstrating that the proportion of suicides to population also increases. The proportion of suicides in Paris, relatively to those in the departments, is considerably greater; and M. de Boismont has sought to show, by an elaborate analysis, that the influence of Paris and Marseilles radiates into the surrounding country, raising the proportion of suicides in the districts nearest to these great centres.

The facts collected by Dr. Bingham show 184 cases of suicide, in 1844, in America. Out of 172 cases in which the season is indicated, 104 were committed in the hot months. The principal mode of death was hanging. It is stated by Leuret and M. Boudin that suicide is far more frequent amongst the black population than the white; and Dr. Baly has shown that violent deaths are twice as frequent amongst the blacks of New York as amongst the whites.

In Belgium there are recorded 620 suicides during the four years from 1835 to 1838, the whole population being 4,260,631.

In Prussia, on the authority of M. Morel de Maréville, there occurred 15,103 suicides in the ten years from 1834 to 1843. In 1843 the population was 15,447,440.

M. Boudin states that in Austria the proportion of voluntary deaths, which stood at 85 in 100,000 of population during the period from

1819 to 1827, rose in the period from 1828 to 1844, to 102 in 100,000.

M. Hübertz, the Danish statist, states that between 1835 and 1844, there occurred in Sweden an average of 407 violent deaths yearly; that in Copenhagen there are about 45 suicides annually.

The figures from Russia are probably not very exact. The following statement, however, by M. Herman, seems to justify the conclusion that the serfs are not so much reconciled to a life of slavery as is sometimes represented. Out of 652 suicides occurring in the western part of the central provinces of the empire in 1821, 458 occurred amongst the serfs; and in the following year, out of 673 suicides, the serfs again figured as 498.

In Mahometan countries suicide is believed to be rare.

M. de Boismont devotes a long and interesting chapter to "the Physiology and Symptomatology of Suicide." In this title he again declares his conviction that suicide is not always a pathological question. He opposes the doctrines of Esquirol, who insisted strongly upon the delirium of suicidists. The truth, as it so often does, lies between the two extremes. *Ni jamais, ni toujours*, is a maxim especially applicable here. M. de Boismont has exceeded by depreciating the share of insanity; M. Esquirol, by assigning to it a too absolute part. We have already said that the possibility of responsible suicide is not to be proved by the indistinct evidence of statistical tables, but by well-observed and well-sifted individual facts. Now, if we apply this test, we shall certainly be struck with the absence of everything indicative of insanity in the conduct of some of the victims—unless, indeed, we regard the act of suicide itself to be such an indication, which is simply begging the question, and placing it beyond the range of serious inquiry. The cases which to our mind prove to demonstration that suicide has been committed by persons of sound mind in the ordinary medical and legal meaning of the term, are those of suicide in companionship, of which our sentimental neighbours present so many singular examples. Two young people encounter an obstacle to their union which appears to them irresistible. In their impatience, they, or rather one of them in the first instance, become disgusted with the world; they discuss between them the project of suicide; their resolution formed, and the preparatory arrangements got up with all the theatrical display conventional on these occasions, they shut themselves in a room, stop up the crevices, light the charcoal braziers, and expire in each other's arms. In some instances the anxiety to be discovered locked in an embrace is so great, that couples have tied themselves together with a shawl, lest in the act of death they should fall asunder! Now, in a deed of this kind there is folly enough, wrongheadedness, and even criminality; but is this insanity? Perhaps yes, in *one* of the lovers—the one that has proposed the mutual suicide, and fascinated his paramour into consent. But it is against the law of probability that *both* were insane.

There are yet other cases which, in this world of struggling interests, disappointments, and conflicting passions, every now and then startle the public by their attendant circumstances. That sharp misery may, in metaphorical language, turn the brain—that the consciousness of guilt, the dread of infamy and punishment, may so overpower the better reason as to suggest self-destruction as the readiest escape from sufferings and the intolerable gaze of the world, are facts too painfully and too frequently illustrated by tragic deeds to admit of doubt. Are we to conclude that, in all the cases of this class, misery, and the sense of guilt and degradation, however fearfully real, have caused insanity, disease? If we admit this, we can scarcely stop at this point; we cannot avoid the ulterior logical conclusion that the same causes, the same passions, have produced insanity—that is, disease—in those cases where, not suicide, but homicide, is the climax. It is to lay down a doctrine for suicide which would infallibly be applied to remove all responsibility for murder. Where is the evidence of insanity in the case of John Sadleir, the gigantic speculator, who at the end of his resources, no longer able to ward off the day of reckoning with his defrauded victims and with society, takes the last fatal resolution, perhaps long contemplated, that ended his career? Does insanity appear in his conduct, or in his preparations for the catastrophe? He had bought the poison beforehand, he had waited for the night, he had walked a long distance to a retired spot, where he might accomplish his purpose without fear of interruption, and be secure from officious help. He had provided himself with a vessel capable of holding an ample draught of the poison; and, should that fail, there was the razor at hand. We see here the stern will of a desperate man, who dreaded life for the shame, degradation, and infamous punishment which was the condition of its preservation. His last letters breathe, indeed, a sentiment of remorse—they declare his incapacity to witness the misery of his victims; but another feeling is also manifest: his career of undetected fraud had touched its end, he no longer hoped to cover the crimes of yesterday by new ones to-morrow; it was the scorn of the world, shame, and infamy, that he could not face. Did any one suspect insanity before he committed suicide? The most trivial circumstances arguing eccentricity or singularity always rise to the memory after the fact, and are ostentatiously elaborated before the coroner's jury into presumptive evidence of insanity. In this case even such evidence is wanting. There is nothing but the act itself to support the presumption of insanity. And let us reflect for a moment upon the consequences into which we should be drawn if we admit that the act of suicide, *per se*, or doubtfully supported, is a proof of insanity. Suppose that John Sadleir had survived his attempt, would the proof of insanity have been weakened? Not one iota. Esquirol has shown that one-half the attempts at suicide fail. He must, then, have been acquitted if put upon his trial for his commercial crimes. A step further, a step from which there is no logical escape: suppose another man, steeped in crime, and feeling the hand of justice upon him; he has but to simulate an attempt at suicide to turn the wrath of society into compassion, and pass from the gaol into the asylum. Unless, then, we are prepared to surrender every safeguard of society—to destroy responsibility altogether, we must be jealous how far we carry the materialist doctrine of insanity.

We have taken some pains to show that M. de Boismont has very much underrated the influence of disease. It is an error as great, a danger as fearful, to exaggerate it.

In taking leave for the present of this strangely fascinating and deeply important subject, we feel ourselves called upon to offer some apologies to the able author of the work before us for the omission of more detailed reference to many topics which he has handled with remarkable vigour and effect. We have been tempted, on several occasions, to offer our own commentaries upon the questions suggested in the text, rather than to attempt a task in which we could scarcely hope to succeed—namely, that of presenting a full and clear analysis of the abundant matter collected by M. de Boismont. For the most ample collection of facts—for the most methodical digest of facts and opinions illustrating the history, nature, social and medico-legal relations of suicide, we refer our readers to the book itself. There is one part of the work to which we would especially invite the attention of our medical friends who are engaged in the treatment of the insane—we mean that in which the author details his method and experience in the treatment of patients afflicted with suicidal tendencies. It is at once the most practical, and that in which M. de Boismont's great experience, sagacity, philanthropy, and sound judgment are most conspicuously displayed.

